# Association between rheumatology patients’ beliefs about medicines and treatment adherence

**DOI:** 10.1590/1980-220X-REEUSP-2025-0252en

**Published:** 2026-01-09

**Authors:** Özlem Kardaş Kin, Arzu Güngör Tolasa

**Affiliations:** 1 Gaziantep Islamic Science and Technology University, Gaziantep, Türkiye.; 2 İzmir City Hospital, İzmir, Türkiye.

**Keywords:** Rheumatology, Compliance, Attitude, Medication Adherence, Nursing, Reumatologia, Complacência (Medida de Distensibilidade), Atitude, Adesão à Medicação, Enfermagem

## Abstract

**Objective:**

This study aimed to examine the association between rheumatology patients’ beliefs about medications and treatment adherence, along with related factors.

**Method:**

A descriptive design was employed. The study was conducted in the Rheumatology Polyclinic and Clinic in Türkiye, including 311 patients. Compliance Questionnaire on Rheumatology (CQR) and Beliefs About Medicines Questionnaire (BMQ-T) were used.

**Results:**

Among participants, 72% were female, and the mean age was 47 years. Rheumatoid arthritis was the most frequent diagnosis (30.9%). The average BMQ score was 47.66 ± 9.58, and the average CQR score was 48.21 ± 5.55. All patients were found to be non-adherent. A statistically significant relationship was observed between BMQ and CQR scores (p < 0.05). CQR scores were higher among males and unemployed participants. Additionally, BMQ scores were significantly associated with marital status and medication use duration (p < 0.05). A moderate negative correlation was found between CQR and the BMQ-anxiety subdimension.

**Conclusion:**

Beliefs about medications significantly affect adherence. Increased anxiety concerning medication use is associated with lower treatment adherence.

## RESUMO

**Objetivo:**

Este estudo teve como objetivo examinar a a.ssociação entre as crenças dos pacientes de reumatologia sobre medicamentos e a adesão ao tratamento, juntamente com fatores relacionados.

**Método:**

Foi empregado um design descritivo. O estudo foi conduzido na Policlínica e Clínica de Reumatologia na Turquia, incluindo 311 pacientes. Foram utilizados o *Compliance Questionnaire on Rheumatology* (CQR) e o *Beliefs About Medicines Questionnaire* (BMQ-T).

**Resultados:**

Entre os participantes, 72% eram mulheres e a média de idade era de 47 anos. A artrite reumatoide foi o diagnóstico mais frequente (30,9%). O escore médio do BMQ foi de 47,66 ± 9,58, e o do CQR foi de 48,21 ± 5,55. Todos os pacientes foram considerados não aderentes. Foi observada uma relação estatisticamente significativa entre os escores do BMQ e do CQR (p < 0,05). Os escores do CQR foram maiores entre os participantes do sexo masculino e desempregados. Além disso, os escores do BMQ foram significativamente associados ao estado civil e à duração do uso de medicamentos (p < 0,05). Foi encontrada uma correlação negativa moderada entre o CQR e a subdimensão de ansiedade do BMQ.

**Conclusão:**

Crenças sobre medicamentos afetam significativamente a adesão. O aumento da ansiedade em relação ao uso de medicamentos está associado à menor adesão ao tratamento.

## INTRODUCTION

Medication adherence remains a cornerstone in the effective management of rheumatic diseases, including rheumatoid arthritis (RA), ankylosing spondylitis (AS), systemic lupus erythematosus (SLE), and gout. With the introduction of disease-modifying antirheumatic drugs (DMARDs) and biologics, remarkable progress has been achieved in slowing disease progression and reducing exacerbations. Nevertheless, a substantial proportion of patients fail to adhere to prescribed treatment regimens, which significantly undermines treatment efficacy and increases the risk of morbidity, mortality, and unnecessary healthcare costs^(1,2)^. The World Health Organization (WHO) defines adherence as “the extent to which a person’s behavior coincides with the accepted recommendations of the healthcare provider”, emphasizing its dynamic and multifactorial nature^(3)^.

Beliefs about medicines—including perceived necessity, concerns about side effects, and skepticism regarding long-term benefits—have consistently been shown to shape adherence behaviors in rheumatology populations^(4,5,6)^. In fact, studies indicate that patients’ beliefs and illness perceptions often exert a stronger influence on adherence than sociodemographic or clinical factors^(7,8)^. Negative or ambivalent beliefs may undermine trust in treatment, even in the presence of severe disease activity, and are commonly reinforced by limited communication with healthcare providers, inadequate social support, and fear of adverse effects^(9,10)^.

Recent systematic reviews highlight that many adherence interventions have overlooked patients’ perspectives, limiting their effectiveness^(9)^. To address this gap, models such as the COM-B framework have been applied to better understand the behavioral mechanisms underlying adherence decisions^(1)^. Given the chronic nature of rheumatic diseases and the potential for long-term complications if treatment is inconsistent, it is essential to investigate patients’ beliefs about medications and identify the barriers they face. By understanding these factors, tailored interventions can be developed to promote optimal adherence, improve clinical outcomes, and ensure costeffective healthcare delivery. This study aimed to examine the association between rheumatology patients’ beliefs about medications and treatment adherence, along with related factors.

## METHODS

### Design of Study

This is a descriptive study. Data were collected in the rheumatology clinics and outpatient clinics of a university hospital between March and August 2024.

### Study Design and Sample

The population of the study was based on individuals who received outpatient and inpatient treatment with rheumatological diagnosis in the Rheumatology Outpatient Clinic and Rheumatology Clinic of Izmir Atatürk Training and Research Hospital between March and August 2024. The sample of the study was calculated using a probabilistic multiple regression analysis calculator, and it was determined that at least 278 patients should be reached with an effect size of 1.0 at a 5% Type 1 error level, with 95% power and the number of independent variables in the study (Available from: http://www.openepi.com/SampleSize/SSPropor.htm). At the end of the study, 311 patients were reached.

### Inclusion and Exclusion Criteria

*Inclusion criteria*: Being a rheumatology patient, volunteering to participate in the study, being able to read and write.

*Exclusion criteria*: Not volunteering to participate in the study.

### Data Collection Tools

Data were collected through a face-to-face data collection method (questionnaire). The administration time of the questionnaire was 10 minutes.

*Personal Characteristics Descriptive Questionnaire Form.* It was prepared by the researcher with the literature^(11,12)^. Consisting of 13 questions, the questionnaire includes individual characteristics such as gender, marital status, and disease-related questions such as duration of the disease and duration of drug use.

*Compliance Questionnaire on Rheumatology (CQR-T).* It is a questionnaire developed to assess treatment adherence in rheumatology patients^(13)^, and a validity and reliability study was conducted in Turkish^(14)^. The questionnaire is 4-point Likert type and consists of 19 items. Six of the 19 items (items 4, 8, 9, 11, 12 and 19) are reverse coded and scored as (4 = 1, 3 = 2, 2 = 3, 1 = 4). The CQR-T total score is calculated by subtracting 19 from the sum of all items and dividing the result by 0.57. The total score ranges from 0 (discordant) to 100 (perfect agreement). Non-compliance is defined as a score equal to or less than 80^(15)^. Patients were grouped as compliant and non-compliant using a cut-off score of 80% according to previous studies^(4)^.

*Beliefs about Medicines Questionnaire (BMQ-T).* The validity and reliability of the questionnaire was carried out in Turkish^(12)^ on Behçet’s disease patients. The aim is to measure individuals’ personal views about the medicines prescribed to them and about medicines in general^(16)^. The questionnaire consists of two parts: a specific and a general one. The BMQ-General section includes two four-item subscales: General Harm and General Overuse. The BMQ-Specific section comprises two five-item subscales that assess patients’ beliefs regarding the necessity of medications for managing their illness and their concerns about potential adverse effects of taking those medications. Respondents indicate the extent of their agreement with each statement using a five-point Likert scale, ranging from “strongly agree = 1” to “strongly disagree = 5.” Each subscale is scored independently by summing the item scores, and the mean score is calculated by dividing the total subscale score by the number of items in that subscale, resulting in a value between 1 and 5. Higher scores indicate stronger beliefs in the respective concept. The overall Cronbach’s alpha values for the BMQ-T Specific and BMQ-T General domains were found to be 0.774 and 0.730, respectively. These results indicate that the Turkish version of the BMQ is a reliable instrument^(16)^. Required permissions for its use in this study were obtained in advance.

### Data Analysis

Data analysis was performed using SPSS 25.0 statistical package software. Data were expressed as mean, standard deviation, percentage, median. Normal distribution analysis was performed for the numerically measured data and according to the results of this analysis, parametric tests were used for variables with normal distribution, and non-parametric tests were used for the others^(17,18)^. Spearman correlation coefficient was used to evaluate the relationship between variables. Mann-Whitney U test and Pearson Chi-square test were used to analyze the data. A p value < 0.05 was considered statistically significant.

### Ethical Aspects

Research permission was obtained from the Gaziantep Islamic Science And Technology University Non-Interventional Clinical Research Ethics Committee (decision no. 357.34.05, meeting date: 23.01.2024). Written and verbal informed consent was obtained from all patients.

## RESULTS

The majority (72.0%) of the rheumatology patients participating in the study were female, 69.5% were married, and 69.8% were not working. The most common medical diagnoses were rheumatoid arthritis (30.9%), ankylosing spondylitis (14.8%), and systemic lupus erythematosus (10.3%). The duration of diagnosis was mostly (60.8%) 5 years or more. Considering the duration of medication use, 54.0% had been using medication for 1–5 years; 93.6% of the patients stated that they did not use non-drug methods, while 20 patients used non-drug methods (Table 1). The majority (2.6%) of the patients who used nondrug methods used vitamin supplements. One percent stated that they exercised. Apart from these methods, other methods that are preferred to a lesser extent are ozone therapy, bioresonance, change in nutrition, massaging the painful joint with St. John’s wort oil, cold water, and okra seed.

**Table 1:** Descriptive characteristics of rheumatology patients – İzmir, Türkiye, 2024.

Variables	Number (n)	Percent (%)
*Mean age: X̄ ± S: 47,61 ± 12,777; Min.-Max = 19,0–80,0*		
Gender
Female	224	72.0
Male	87	28.0
Marital status
Married	216	69.5
Single	62	19.9
Widowed/divorced	33	10.6
Employment status
Working	94	30.2
Unemployed	217	69.8
Income status
Income less than expenditure	76	24.4
Income equals expenditure	174	55.9
Income more than expenditure	61	19.6
Medical Diagnosis
Rheumatoid Arthritis	96	30.9
Ankylosing Spondylitis	46	14.8
Systemic Lupus Erythematosus	31	10.3
Spondyloarthropathy	27	8.7
Psoriatic Arthritis	20	6.4
Scleroderma	19	6.1
Familial Mediterranean Fever (FMF)	13	4.2
Sjögren's Syndrome	12	3.9
Illness duration		
3 months-1 year	24	7.7
1–5 years	98	31.5
>5 years	189	60.8
Duration of medication use
1–5 years	168	54.0
5–10 years	94	30.2
>10 years	49	15.8
Use of non-drug methods
Yes	20	6.4
No	291	93.6
Permanent problems in the body
Yes	88	28.3
No	223	71.7
Problem caused by the disease
Wound	10	3.2
Joint deformity	9	2.9
Co-morbid chronic disease
Yes	125	40.2
No	186	59.8
Co-morbid chronic disease *(n = 125)
Hypertension	46	14.8
Diabetes Mellitus + Hypertension	11	3.5
Chronic heart failure	11	3.5
Other diseases	42	13.5
Drug group *(n = 356)
Conventional Synthetic DMARDs	153	43
Biological DMARDs	88	24.7
Corticosteroids	76	21.3
NSAI/others	33	9.2

A total of 28.3% of the participants reported that they had problems related to rheumatological diseases. The most frequently reported condition related to the problem occurring in the body was wound formation (3.2%), followed by joint deformity (2.9%). A total of 40.2% had a concomitant chronic disease. The most common chronic disease in patients with rheumatological diagnosis was hypertension, with 14.8%. Most of the drugs used by patients with rheumatological diagnosis are antineoplastic agents (Actemra, Imuran, Methotrexate etc.). Corticosteroids (Dekort tablets etc.), antimalarial drugs (Plaquenil tablets etc.), and intestinal anti-inflammatory agents (Salozopyrin) are frequently preferred.

The mean total score of Beliefs About Medicines (BMQ) was 47.66 ± 9.58 and the mean total score of Compliance Questionnaire on Rheumatology (CQR) was 48.21 ± 5.55. A statistically significant relationship was found between beliefs about medications and adherence to treatment (p < 0.005) (Table 2).

**Table 2:** Distribution of Beliefs about Medicines (BMQ) and Compliance Questionnaire on Rheumatology (CQR) Total Mean Scores – İzmir, Türkiye, 2024.

Scales	Min–Max.	X ± SS	Cronbach’s Alfa
BMQ	18–79	47.66 ± 9.58	.001
CQR	30–64	48.21 ± 5.55	.000

A weak, negative and significant relationship was found between adherence to treatment and beliefs about drugs. Beliefs about medications had a negative effect on adherence to treatment (Table 3).

**Table 3:** Relationship between Beliefs about Medicines and Adherence to Treatment – İzmir, Türkiye, 2024.

	BMQ
CQR	
Pearson r	-.216^*^
p	.000
n	311

*Pearson Correlation Analysis. p < 0.05.

Correlation is significant at p < 0.01 level.

According to the results of the statistical analysis, a statistically significant relationship was found between the duration of drug use and beliefs about drugs (p < 0.05). As the duration of drug use increases, beliefs about drugs increase. However, no significant relationship was found between the duration of drug use and adherence to treatment (p > 0.05). No significant correlation was found between the duration of diagnosis and beliefs about drugs and rheumatology adherence (p > 0.05) (Table 4).

**Table 4:** Comparison of the Effect of Disease Related Characteristics on Beliefs about Medicines and adherence to treatment – İzmir, Türkiye, 2024.

Variables		BMQ				CQR		
		N	Mean Rank	d.f./Z	Test statistics p value	Mean Rank	d.f./Z	Test statistics p value
Diagnosis time	3 months – 1 year	24	148.60	d.f.: 2	KW: 5.657^*^	178.63	d.f.: 2	KW: 2.105^*^
	1–5 years	98	139.38		p: .059	159.06		p: .349
	>5 years	189	165.56			151.54		
Duration of medication	1–5 years	168	141.93	d.f.: 2	KW: 9.470^*^	155.39	d.f.: 2	KW: .033^*^
	5–10 years	94	168.68		**p: 009**	157.41		p: .983
	>10 years	49	179.93			155.37		
Gender	Woman	224	159.53	Z: -1.112	MU: 8953.500^**^ p: 266	149.44	Z: -2.067	MU: 8275.500^**^ **p: .039**
	Male	87	146.91			172.88	
Employment status	Working	94	170.53	Z: -1.878	MU: 8833.000^**^ p: 0.060	135.65	Z: -2.632	MU: 8286.000^**^ **p: 0.008**
	Not working	217	149.71			164.82	
Marital status	Married	216	156.50	d.f.: 2	KW: 6.387^*^	159.72	d.f.: 2	KW: 3.628^*^
	Single	62	137.90		**p: .041**	137.10		p: .163
	Divorced/widowed	33	186.74			167.14		
Using non-drug methods	Yes	20	183.05	Z: -1.392	MU: 2369.000^**^ p: .164	127.00	Z: -1.494	MU: 2330.000^**^ p: .135
	No	291	154.14			157.99	
Persistent condition in the body	Yes	88	153.92	Z: -.256	MU: 9629.000^**^ p: .798	146.73	Z: -1.145	MU: 8996.000^**^
	No	223	156.82			159.66		p: .252
Chronic Disease	Yes	125	161.86	Z: -.943	MU: 10892.50^**^ p: .346	163.67	Z: -1.236	MU: 10666.000^**^ p: .217
	No	186	152.06			150.84	
Diagnosis	RA	96	138.85	d.f.: 10	KW: 25.026^*^	128.23	d.f.: 10	KW: 15.763^*^ p: .107
	AS	46	172.36		**p: .005**	151.77	
	SLE	32	141.58			146.56		
	Spondyloarthropathy	27	128.11			167.63		
	Psoriatic Arthritis	20	179.00			156.48		
	Scleroderma	26	131.46			168.75		
	FMF	13	138.15			143.50		

p < 0,05.

^*^KW: Kruskal Wallis H Test;

^**^MU: Mann-Whitney U Test.

While there was a significant difference in adherence to treatment according to gender (p < 0.05), there was no significant difference in beliefs about drugs according to gender (p > 0.05). It is observed that adherence to treatment is higher in men. No significant correlation was found between the use of non-drug methods, permanent condition in the body, and additional chronic disease and beliefs about drugs and adherence to treatment (p > 0.05) (Table 4).

A statistically significant difference was found between medical diagnosis and adherence to treatment (p < 0.05). While there was a significant difference between adherence to treatment according to employment status (p < 0.05), no significant difference was found between beliefs about medications according to employment status (p > 0.05); and although there was a significant difference in beliefs about drugs according to marital status (p < 0,05), no significant difference was found between adherence to treatment and marital status (p > 0,05). The score of divorced/widowed people was found to be higher (Table 4).

It was determined that there was a moderate negative correlation between the CRQ and anxiety about the use of medicines. As the individual’s level of concern about the use of medication increases, his/her adherence decreases (Table 5).

**Table 5:** Correlation analysis between Beliefs About Medicines Questionnaire scores and Compliance Questionnaire on Rheumatology Scores – İzmir, Türkiye, 2024.

	CQR score	
	r	p
BMQ-T Specific Necessity^*^	-.337^*^	.096
BMQ-T Specific Concerns^*^	-.094	**.000**
BMQ-T General Overuse^*^	.042	.098
BMQ-T General Harm^*^	-.094	.466

r = Spearman correlation test *median (25th–75th percentile).

CQR: Compliance Questionnaire on Rheumatology; BMQ-T: Turkish Beliefs About Medicines Questionnaire.

In summary, rheumatology patients’ beliefs about medications negatively affected adherence, with gender, marital status, and employment status influencing adherence and beliefs, longer medication use associated with stronger beliefs, and higher medication-related anxiety linked to lower adherence, highlighting the complex relationship between patient characteristics, beliefs, and adherence in rheumatology care.

## DISCUSSION

In the study, it was determined that the duration of diagnosis was mostly (60.8%) 5 years or more. In the study conducted in Spain^(19)^, it was found that the average duration of the disease was 8 years. Similarly, in the study carried out in Türkiye^(4)^, the average observed duration of the disease was 8 years. Since rheumatological diseases are chronic, it is a known fact that patients have been diagnosed a long time before.

In our study, it was found that 40.2% of the patients had an accompanying chronic disease, and the most common was hypertension (14.8%). In parallel with our finding, 31.9% of the patients reported additional chronic diseases in the study by Omair et al.^(20)^. Hypertension (20.2%) and diabetes mellitus (17.2%) were among the most common comorbidities.

In the study, it was observed that there was a significant difference in adherence to treatment according to gender, and adherence was higher in men. Similar to our finding, a study conducted on rheumatoid arthritis (RA) patients in Nigeria^(12)^ reported that male gender was associated with adherence. Unlike these findings, a study on RA patients in Kerman-Shah, Iran^(21)^, reported that gender was not associated with treatment adherence. Menopause and stressful situations are known to cause hormonal changes in women. In addition, female hormonal factors play an important role in the pathogenesis of RA^(22)^. Fluctuations in estrogen and progesterone levels may influence both disease activity and psychological status, leading to fatigue, altered pain perception, and mood changes^(23)^. These physiological and psychosocial effects can reduce adherence to complex treatment regimens. Moreover, since female hormones play a significant role in the pathogenesis and severity of RA, they may indirectly affect treatment adherence^(24)^. Therefore, the higher adherence observed in men in our study can partly be explained by these factors.

A statistically significant difference was found between medical diagnosis and adherence to treatment (p < 0.05). In a master’s thesis in Türkiye^(11)^, it is seen that 63% of individuals with scleroderma have a medium level of treatment adherence according to the ‘Morisky Treatment Adherence Scale’. It was determined that the medication adherence of patients with Familial Mediterranean Fever was at a good level^(25)^. The diversity of the scale and medical diagnosis used to measure adherence affects the individual’s adherence status evaluation.

In the study conducted in Saudi RA patients, being unemployed was associated with better adherence^(20)^. This finding is similar to that of our study. In this study, the rheumatology adjustment score of unemployed patients was higher than that of employed patients. It can be said that non-employed people have higher adherence to treatment due to situations such as time spent at work, environmental factors, forgetting to take medication due to work intensity, and work stress.

The mean total score of the CQR-T was found to be 48.21 ± 5.55. All patients included in the study were determined as non-compliant (CQR score ≤80%), because according to CQR, non-adherence is defined as a score equal to or less than 80^(13,15)^. Another study conducted in Türkiye^(15)^ found the mean score of the CQR to be 67.4 ± 11.7 in patients with familial Mediterranean fever. In this study, the rate of non-adherent patients according to CQR was determined as 83.8%. In the study of patients with Ankylosing Spondylitis, the mean CQR score was 73.59^(4)^. A total of 64.6% were evaluated as nonadherent (CQR score ≤80%), while 35.4% were evaluated as concordant (CQR score >80%). In a study on a rheumatoid arthritis population in Kenya^(26)^, the mean score of CQR-5 was determined as 79% and patients’ adherence was found to be 49.5%. When analyzing the literature, different scales have been used to assess adherence in rheumatology patients. One of them is Morisky Medication Adherence Scale^(27)^. While this scale assesses adherence to treatment, the CQR is a questionnaire specifically developed to assess adherence to disease treatment in individuals with rheumatologic diseases^(13)^. In this study, more than one rheumatological diagnosis group was included in the scope, and a tertiary care center was used to collect data.

No significant relationship was found between the duration of diagnosis and adherence to treatment in this study (p > 0.05). However, it was found that the duration of the disease contributed significantly to higher adherence measures^(12)^. It is thought that the difference in medical diagnosis affects this situation.

In the study of patients diagnosed with RA^(28)^, it was found that deterioration in functional status and pain that occurred with the increase in the number of chronic diseases negatively affected adherence to treatment. In this study, no significant correlation was found between the use of non-pharmacological methods, permanent damage to the body, and additional chronic diseases and beliefs about medications and adherence to treatment.

No significant difference was found between marital status, duration of disease, duration of drug use, use of non-drug methods, permanent status in the body, additional chronic disease, and adherence to treatment. Similar findings were found in other studies^(21)^.

In this study, the BMQ mean total score was found to be 47.66 ± 9.58. In a study of RA patients in Iraq^(29)^, the BMQ total score was determined as 59.3 ± 6.9.

It was seen that as the duration of drug use increased, the general harm belief about drugs decreased and patients became more familiar with their treatments^(29)^. In this study, a statistically significant relationship was found between the duration of drug use and beliefs about drugs. As the duration of drug use increases, the belief status increases.

Determining attitudes and beliefs about treatment are very important to ensure adherence to treatment. In this study, a weak, negative, and significant correlation was found between CQR and BMQ. When the sub-dimensions were analyzed, it was determined that there was a moderate negative correlation between CRQ and concern about the use of drugs. As the individuals’ level of concern about the use of medication increases, their adherence decreases. Similarly, in Türkiye, a negative correlation between BMQ and CQR scores was found; as the belief that colchicine is necessary increases, adherence increases^(15)^. In addition, it was showed that adherence was significantly associated with the belief of necessity and concerns about drug treatment^(30)^. A significant correlation between CQR and BMQ score was found in a study conducted in Morocco^(31)^. The study on Behçet’s patients found that, on the contrary, all BMQ subscales, excluding the general-overuse score, showed a strong and significant correlation with the CQR^(32)^. In summary, these studies show that beliefs about the necessity and harm of medication and concerns about side effects are predictors of adherence. According to the findings of our study, as the individuals’ concern about medication use increases, it has an inverse effect on their adherence to treatment.

Our results highlight the crucial importance of incorporating patients’ medication-related beliefs and concerns into standard rheumatology care. Nurses and other healthcare professionals should employ patient-centered communication strategies that explore and address misconceptions, worries, and anxieties about long-term medication use. Educational programs tailored to patients’ sociodemographic characteristics—such as gender, marital status, and employment status—may improve adherence by reducing concerns about side effects and reinforcing the perceived necessity of treatment^(33)^. At the policy level, the early identification of patients at risk of non-adherence may be strengthened by integrating medication-belief screening and psychosocial assessments into routine rheumatology care pathways.

From an occupational health perspective, workplace-based health support programs and flexible medication management plans may be beneficial, as employed individuals often face greater barriers to adherence due to time constraints and work-related stress. Evidence suggests that integrated workplace health and pharmacy services significantly improve medication adherence compared with community-based services^(34)^. These implications are particularly relevant for rheumatology practice in Türkiye, where the burden of chronic diseases is high and work-related factors may influence adherence patterns.

### Limitations of the Study

A notable limitation of this study is its single-center design, conducted within a tertiary care hospital. This may constrain the generalizability of the findings to broader clinical practice settings or more heterogeneous rheumatology populations, as patient demographics, resource availability, and institutional protocols in tertiary centers may differ from those in community hospitals or other healthcare environments. Psychological disorders such as depression or anxiety were not analyzed. Since assessing the presence of depression and anxiety may affect the concepts of illness adaptation and beliefs, their evaluation would enrich the content of the study to obtain more comprehensive information.

## CONCLUSION

The individual’s beliefs about the disease and the medications used have a very important place in the evaluation of adherence to treatment related to chronic diseases. To support the relationship between beliefs and compliance, the individual should be evaluated in the socio-psychological dimension. Gender, marital status, employment status, and duration of medication use are among the parameters that need to be considered in terms of adherence. Concern about the drug affects the individual’s adherence. How conscious the individual is about the use of medication must be evaluated by both the physician and the nurse. The effect, side effects, dosage, and frequency of use of drugs should be questioned, and this information should be included in patient education.

## Data Availability

The entire dataset supporting the results of this study is available upon request to the corresponding author.
