# Supplementary material for: Association between rheumatology patients’ beliefs about medicines and treatment adherence
Source: Rev Esc Enferm USP. 2026 Jan 9;59:e20250252. doi: 10.1590/1980-220X-REEUSP-2025-0252en (PMC12788722; doi:10.1590/1980-220X-REEUSP-2025-0252en)
Supplement: Translation

# Translation: Asociación entre las creencias de los pacientes de reumatología sobre los medicamentos y la adherencia al tratamiento

**DOI:** 10.1590/1980-220X-REEUSP-2025-0252es

**Authors:** Özlem Kardaş Kin, Arzu Güngör Tolasa

**Affiliations:** 1 Gaziantep Islamic Science and Technology University, Gaziantep, Türkiye.; 2 İzmir City Hospital, İzmir, Türkiye.

**Keywords:** Reumatología;, Adaptabilidad, Actitud, Cumplimiento de la Medicación, Enfermería

## Abstract

**Objetivo:**

Este estudio tuvo como objetivo examinar la asociación entre las creencias de los pacientes de reumatología sobre los medicamentos y la adherencia al tratamiento, junto con otros factores relacionados.

**Método:**

Se empleó un diseño descriptivo. El estudio se realizó en la Policlínica y Clínica de Reumatología de Turquía e incluyó a 311 pacientes. Se utilizaron el *Compliance Questionnaire on Rheumatology* (CQR) y *Beliefs About Medicines Questionnaire* (BMQ-T).

**Resultados:**

Entre los participantes, el 72% eran mujeres y la edad media era de 47 años. La artritis reumatoide fue el diagnóstico más frecuente (30,9%). La puntuación media del BMQ fue de 47,66 ± 9,58 y la puntuación media del CQR fue de 48,21 ± 5,55. Se encontró que todos los pacientes no cumplían con el tratamiento. Se observó una relación estadísticamente significativa entre las puntuaciones BMQ y CQR (p < 0,05). Las puntuaciones CQR fueron más altas entre los hombres y los participantes desempleados. Además, las puntuaciones del BMQ se asociaron significativamente con el estado civil y la duración del uso de medicamentos (p < 0,05). Se encontró una correlación negativa moderada entre el CQR y la subdimensión ansiedad del BMQ.

**Conclusión:**

Las creencias sobre los medicamentos afectan significativamente la adherencia. El aumento de la ansiedad relacionada con el uso de medicamentos se asocia con una menor adherencia al tratamiento.

## INTRODUCCIÓN

La adherencia a la medicación sigue siendo una piedra angular en el tratamiento eficaz de las enfermedades reumáticas, como la artritis reumatoide (AR), la espondilitis anquilosante (EA), el lupus eritematoso sistémico (LES) y la gota. Con la introducción de los fármacos antirreumáticos modificadores de la enfermedad (FAME) y los biológicos, se han logrado avances notables en la ralentización de la progresión de la enfermedad y la reducción de las exacerbaciones. No obstante, una proporción considerable de pacientes no sigue los regímenes de tratamiento prescritos, lo que socava significativamente la eficacia del tratamiento y aumenta el riesgo de morbilidad, mortalidad y costos sanitarios innecesarios^(1,2)^. La Organización Mundial de la Salud (OMS) define la adherencia como “el grado en que el comportamiento de una persona coincide con las recomendaciones aceptadas del profesional sanitario”, haciendo hincapié en su naturaleza dinámica y multifactorial^(3)^.

Se ha demostrado sistemáticamente que las creencias sobre los medicamentos —incluida la percepción de su necesidad, la preocupación por los efectos secundarios y el escepticismo sobre sus beneficios a largo plazo—determinan los comportamientos de adherencia en las poblaciones reumatológicas^(4,5,6)^. De hecho, los estudios indican que las creencias y percepciones de los pacientes sobre la enfermedad suelen ejercer una influencia mayor en la adherencia que los factores sociodemográficos o clínicos^(7,8)^. Las creencias negativas o ambivalentes pueden socavar la confianza en el tratamiento, incluso en presencia de una actividad grave de la enfermedad, y suelen verse reforzadas por una comunicación limitada con los proveedores de atención médica, un apoyo social inadecuado y el temor a los efectos adversos^(9,10)^.

Revisiones sistemáticas recientes destacan que muchas intervenciones de adherencia han pasado por alto las perspectivas de los pacientes, lo que limita su eficacia^(9)^. Para abordar esta laguna, se han aplicado modelos como el marco COM-B (Capability, Opportunity and Motivation to Behavior) para comprender mejor los mecanismos conductuales que subyacen a las decisiones de adherencia^(1)^. Dada la naturaleza crónica de las enfermedades reumáticas y la posibilidad de complicaciones a largo plazo si el tratamiento es inconsistente, es esencial investigar las creencias de los pacientes sobre los medicamentos e identificar las barreras a las que se enfrentan. Al comprender estos factores, se pueden desarrollar intervenciones personalizadas para promover una adherencia óptima, mejorar los resultados clínicos y garantizar una prestación de asistencia sanitaria rentable. El objetivo de este estudio fue examinar la asociación entre las creencias de los pacientes reumatológicos sobre los medicamentos y la adherencia al tratamiento, junto con los factores relacionados.

## MÉTODOS

### Diseño del Estudio

Se trata de un estudio descriptivo. Los datos se recolectaron para recolectar en las clínicas reumatológicas y ambulatorias de un hospital universitario entre marzo y agosto de 2024.

### Diseño del Estudio y Muestra

La población del estudio se basó en personas que recibieron tratamiento ambulatorio y hospitalario con diagnóstico reumatológico en la Clínica Ambulatoria de Reumatología y la Clínica de Reumatología del Hospital de Formación e Investigación Atatürk de Izmir entre marzo y agosto de 2024. La muestra del estudio se calculó utilizando una calculadora de análisis de regresión múltiple probabilística, y se determinó que se debía llegar al menos a 278 pacientes con un tamaño del efecto de 1,0 a un nivel de error del 5% de tipo 1, con una potencia del 95% y el número de variables independientes del estudio (disponible en: http://www.openepi.com/SampleSize/SSPropor.htm). Al final del estudio, se llegó a 311 pacientes.

### Criterios de Inclusión y Exclusión

*Criterios de inclusión*: Ser paciente de reumatología, ofrecerse voluntario para participar en el estudio, saber leer y escribir.

*Criterios de exclusión*: No ofrecerse voluntario para participar en el estudio.

### Herramientas de Recopilación de Datos

Los datos se recopilaron mediante un método de recopilación de datos presencial (cuestionario). El tiempo de administración del cuestionario fue de 10 minutos.

*Cuestionario descriptivo de características personales.* Fue elaborado por el investigador con la bibliografía^(11,12)^. El cuestionario, que consta de 13 preguntas, incluye características individuales como el sexo, el estado civil y preguntas relacionadas con la enfermedad, como la duración de la misma y la duración del uso de medicamentos.

*Cuestionario de cumplimiento en reumatología* (CQR-T). Se trata de un cuestionario elaborado para evaluar el cumplimiento del tratamiento en pacientes reumáticos^(13)^, y se ha realizado un estudio de validez y fiabilidad en turco^(14)^. El cuestionario es de tipo Likert de 4 puntos y consta de 19 ítems. Seis de los 19 ítems (ítems 4, 8, 9, 11, 12 y 19) están codificados de forma inversa y se puntúan como (4 = 1, 3 = 2, 2 = 3, 1 = 4). La puntuación total del CQR-T se calcula restando 19 de la suma de todos los ítems y dividiendo el resultado por 0,57. La puntuación total oscila entre 0 (discordancia) y 100 (acuerdo perfecto). El incumplimiento se define como una puntuación igual o inferior a 80^(15)^. Los pacientes se agruparon en cumplidores y no cumplidores utilizando una puntuación de corte del 80% según estudios previos^(4)^.

*Cuestionario de creencias sobre los medicamentos* (BMQ-T). La validez y fiabilidad del cuestionario se llevó a cabo en turco^(12)^ en pacientes con enfermedad de Behçet. El objetivo es medir las opiniones personales de los individuos sobre los medicamentos que se les recetan y sobre los medicamentos en general^(16)^. El cuestionario consta de dos partes: una específica y otra general. La sección BMQ-General incluye dos subescalas de cuatro ítems: Daño general y Uso excesivo general. La sección BMQ-Específica comprende dos subescalas de cinco ítems que evalúan las creencias de los pacientes sobre la necesidad de los medicamentos para controlar su enfermedad y sus preocupaciones sobre los posibles efectos adversos de tomarlos. Los encuestados indican su grado de acuerdo con cada afirmación utilizando una escala Likert de cinco puntos, que va desde “totalmente de acuerdo = 1” a “totalmente en desacuerdo = 5”. Cada subescala se puntúa de forma independiente sumando las puntuaciones de los ítems, y la puntuación media se calcula dividiendo la puntuación total de la subescala por el número de ítems de esa subescala, lo que da como resultado un valor entre 1 y 5. Las puntuaciones más altas indican una mayor creencia en el concepto respectivo. Los valores generales del alfa de Cronbach para los dominios específicos y generales del BMQ-T fueron de 0,774 y 0,730, respectivamente. Estos resultados indican que la versión turca del BMQ es un instrumento fiable^(16)^. Se obtuvieron por adelantado los permisos necesarios para su uso en este estudio.

### Análisis de Datos

El análisis de datos se realizó utilizando el paquete de software estadístico SPSS 25.0. Los datos se expresaron como media, desviación estándar, porcentaje y mediana. Se realizó un análisis de distribución normal para los datos medidos numéricamente y, según los resultados de este análisis, se utilizaron pruebas paramétricas para las variables con distribución normal y pruebas no paramétricas para las demás^(17,18)^. Se utilizó el coeficiente de correlación de Spearman para evaluar la relación entre las variables. Se utilizaron la prueba U de Mann-Whitney y la prueba chi-cuadrado de Pearson para analizar los datos. Se consideró estadísticamente significativo un valor p < 0,05.

### Aspectos Éticos

Se obtuvo el permiso para la investigación del Comité de Ética en Investigación Clínica No Intervencionista de la Universidad Islámica de Ciencia y Tecnología de Gaziantep (decisión n.º 357.34.05, fecha de la reunión: 23.01.2024). Se obtuvo el consentimiento informado por escrito y verbal de todos los pacientes.

## RESULTADOS

La mayoría (72,0%) de los pacientes reumatológicos que participaron en el estudio eran mujeres, el 69,5% estaban casados y el 69,8% no trabajaban. Los diagnósticos médicos más comunes fueron la artritis reumatoide (30,9%), la espondilitis anquilosante (14,8%) y el lupus eritematoso sistémico (10,3%). La duración del diagnóstico fue en su mayoría (60,8%) de 5 años o más. Teniendo en cuenta la duración del uso de medicamentos, el 54,0% había estado utilizando medicamentos durante 1-5 años; el 93,6% de los pacientes afirmaron que no utilizaban métodos no farmacológicos, mientras que 20 pacientes utilizaban métodos no farmacológicos (Tabla 1). La mayoría (2,6%) de los pacientes que utilizaban métodos no farmacológicos tomaban suplementos vitamínicos. El 1% afirmó que hacía ejercicio. Aparte de estos métodos, otros métodos menos utilizados son la ozonoterapia, la biorresonancia, el cambio en la alimentación, el masaje de la articulación dolorida con aceite de hipérico, el agua fría y las semillas de okra.

**Tabla 1:** Características descriptivas de los pacientes reumatológicos – Esmirna, Turquía, 2024.

Variables	Número (n)	Porcentaje (%)
*Edad media: X̄ ± S: 47,61± 12,777; Mín.-Máx. = 19,0–80,0*		
Género
Mujer	224	72,0
Hombres	87	28,0
Estado civil
Casado	216	69,5
Soltero	62	19,9
Viudo/divorciado	33	10,6
Situación laboral		
Trabajando	94	30,2
Desempleado	217	69,8
Situación económica		
Ingresos inferiores a los gastos	76	24,4
Ingresos iguales a los gastos	174	55,9
Los ingresos superan a los gastos Diagnóstico médico	61	19,6
Diagnóstico médico		
Artritis reumatoide	96	30,9
Espondilitis anquilosante	46	14,8
Lupus eritematoso sistémico	31	10,3
Espondiloartropatía	27	8,7
Artritis psoriásica	20	6,4
Esclerodermia	19	6,1
Fiebre mediterránea familiar (FMF)	13	4,2
Síndrome de Sjögren	12	3,9
Duración de la enfermedad		
3 meses-1 año	24	7,7
1–5 años	98	31,5
> 5 años	189	60,8
Duración del uso de medicamentos		
1–5 años	168	54,0
5–10 años	94	30,2
10 años	49	15,8
Uso de métodos no farmacológicos		
Sí	20	6,4
No	291	93,6
Problemas permanentes en el cuerpo		
Sí	88	28,3
No	223	71,7
Problema causado por la enfermedad		
Herida	10	3,2
Deformidad articular	9	2,9
Enfermedad crónica comórbida		
Sí	125	40,2
No	186	59,8
Enfermedad crónica comórbida *(n = 125)		
Hipertensión	46	14,8
Diabetes mellitus + hipertensión	11	3,5
Insuficiencia cardíaca crónica	11	3,5
Otras enfermedades	42	13,5
Grupo de fármacos ^*^(n = 356)		
Fármacos antirreumáticos modificadores de la enfermedad (FAME) sintéticos convencionales	153	43
Fármacos antirreumáticos modificadores de la enfermedad biológicos	88	24,7
Corticoesteroides	76	21,3
AINE/otros	33	9,2

Un total del 28,3% de los participantes informaron que tenían problemas relacionados con enfermedades reumatológicas. La afección relacionada con el problema que se presentaba en el cuerpo que se informó con mayor frecuencia fue la formación de heridas (3,2%), seguida de la deformidad articular (2,9%). Un total del 40,2% tenía una enfermedad crónica concomitante. La enfermedad crónica más común en los pacientes con diagnóstico reumatológico fue la hipertensión, con un 14,8%. La mayoría de los medicamentos utilizados por los pacientes con diagnóstico reumatológico son agentes anti-neoplásicos (Actemra, Imuran, metotrexato, etc.). Se prefieren con frecuencia los corticosteroides (comprimidos de Dekort, etc.), los medicamentos contra la malaria (comprimidos de Plaquenil, etc.) y los agentes antiinflamatorios intestinales (Salozopyrin).

La puntuación media total del cuestionario sobre creencias acerca de los medicamentos (BMQ) fue de 47,66 ± 9,58 y la puntuación media total del cuestionario sobre cumplimiento terapéutico en reumatología (CQR) fue de 48,21 ± 5,55. Se encontró una relación estadísticamente significativa entre las creencias acerca de los medicamentos y el cumplimiento del tratamiento (p < 0,005) (Tabla 2).

**Table 2:** Distribución de las puntuaciones medias totales del cuestionario sobre creencias acerca de los medicamentos (BMQ) y del cuestionario sobre cumplimiento terapéutico en reumatología (CQR) – Esmirna, Turquía, 2024.

Escalas	Mín.-Máx.	X ± SS	Alfa de Cronbach
BMQ	18–79	47,66 ± 9,58	.001
CQR	30–64	48,21 ± 5,55	0,000

Se encontró una relación débil, negativa y significativa entre la adherencia al tratamiento y las creencias sobre los medicamentos. Las creencias sobre los medicamentos tuvieron un efecto negativo en la adherencia al tratamiento (Tabla 3).

**Table 3:** Relación entre las creencias sobre los medicamentos y la adherencia al tratamiento – Esmirna, Turquía, 2024.

	BMQ
CQR	
Pearson r	-.216^*^
p	.000
n	311

*Análisis de correlación de Pearson. p < 0,05.

La correlación es significativa a un nivel p < 0,01.

Según los resultados del análisis estadístico, se encontró una relación estadísticamente significativa entre la duración del consumo de drogas y las creencias sobre las drogas (p < 0,05). A medida que aumenta la duración del consumo de drogas, aumentan las creencias sobre las drogas. Sin embargo, no se encontró una relación significativa entre la duración del consumo de drogas y la adherencia al tratamiento (p > 0,05). No se encontró una correlación significativa entre la duración del diagnóstico y las creencias sobre las drogas y la adherencia a la reumatología (p > 0,05) (Tabla 4).

**Table 4:** Comparación del efecto de las características relacionadas con la enfermedad en las creencias sobre los medicamentos y la adherencia al tratamiento – Esmirna, Turquía, 2024.

Variables		BMQ				CQR		
		N	Rango medio	d.f./Z	Estadísticas de prueba Valor p	Rango medio	d.f./Z	Estadísticas de prueba Valor p
Tiempo de diagnóstico	3 meses – 1 año	24	148,60	d.f.: 2	KW: 5,657^*^	178,63	d.f.: 2	KW: 2,105^*^
	1–5 años	98	139,38		p: .059	159,06		p: .349
	>5 años	189	165,56			151,54		
Duración de la medicación	1–5 años	168	141,93	d.f.: 2	KW: 9,470^*^	155,39	d.f.: 2	KW: .033^*^
	5–10 años	94	168,68		**p: 009**	157,41		p: 0,983
	>10 años	49	179,93			155,37		
Género	Mujer	224	159,53	Z: -1,112	MU: 8953,500^**^	149,44	Z: -2.067	MU: 8275,500^**^
	Masculino	87	146,91		p: 266	172,88		**p: 0,039**
Situación laboral	Trabajando	94	170,53	Z: -1,878	MU: 8833,000^**^	135,65	Z: -2.632	MU: 8286,000^** **p: 0,008**^
	No funciona	217	149,71			164,82	
Estado civil	Casado	216	156,50	d.f.: 2	KW: 6.387^*^	159,72	d.f.: 2	KW: 3,628^*^
	Único	62	137,90		**p: .041**	137,10		p: .163
	Divorciado/viudo	33	186,74			167,14		
Uso de métodos no farmacológicos	Sí	20	183,05	Z: -1,392	MU: 2369,000^**^	127,00	Z: -1.494	MU: 2330,000^**^ p: .135
	No	291	154,14		p: .164	157,99	
Afección persistente en el organismo	Sí	88	153,92	Z: -0,256	MU: 9629,000^**^ p: .798	146,73	Z: -1.145	MU: 8996,000^**^
	No	223	156,82		159,66		p: .252
Enfermedad crónica	Sí	125	161,86	Z: -0,943	MU: 10892,50^**^ p: .346	163,67	Z: -1.236	MU: 10666,000^**^ p: .217
	No	186	152,06			150,84	
Diagnóstico	AR	96	138,85	d.f.: 10	KW: 25,026^*^	128,23	d.f.: 10	KW: 15,763^*^ p: .107
	AS	46	172,36		**p: .005**	151,77	
	SLE	32	141,58			146,56		
	Espondilo artropatía	27	128,11			167,63		
	Artritis psoriásica	20	179,00			156,48		
	Esclerodermia	26	131,46			168,75		
	FMF	13	138,15			143,50		

p < 0,05.

*KW: prueba H de Kruskal Wallis;

**MU: prueba U de Mann-Whitney.

Si bien se observó una diferencia significativa en la adherencia al tratamiento según el sexo (p < 0,05), no se observó una diferencia significativa en las creencias sobre los medicamentos según el sexo (p > 0,05). Se observa que la adherencia al tratamiento es mayor en los hombres. No se encontró ninguna correlación significativa entre el uso de métodos no farmacológicos, la condición permanente en el organismo y las enfermedades crónicas adicionales, y las creencias sobre los medicamentos y la adherencia al tratamiento (p > 0,05) (Tabla 4).

Se encontró una diferencia estadísticamente significativa entre el diagnóstico médico y la adherencia al tratamiento (p < 0,05). Si bien hubo una diferencia significativa entre la adherencia al tratamiento según la situación laboral (p < 0,05), no se encontró ninguna diferencia significativa entre las creencias sobre los medicamentos según la situación laboral (p > 0,05); y aunque hubo una diferencia significativa en las creencias sobre los medicamentos según el estado civil (p < 0,05), no se encontró ninguna diferencia significativa entre el cumplimiento del tratamiento y el estado civil (p > 0,05). Se observó que la puntuación de las personas divorciadas/viudas era más alta (Tabla 4).

Se determinó que existía una correlación negativa moderada entre el CRQ y la ansiedad sobre el uso de medicamentos. A medida que aumenta el nivel de preocupación del individuo sobre el uso de medicamentos, disminuye su adherencia (Tabla 5).

**Table 5:** Tabla 5 – Análisis de correlación entre las puntuaciones del Cuestionario de Creencias sobre los Medicamentos y las puntuaciones del Cuestionario de Adherencia en Reumatología – Esmirna, Turquía, 2024.

	Puntuación del CQR	
	r	p
BMQ-T Necesidad específica^*^	-0,337^*^	0,096
Preocupaciones específicas de BMQ-T^*^	-0,094	**.000**
BMQ-T Uso excesivo general^*^	0,042	0,098
BMQ-T Daño general^*^	-0,094	0,466

r = prueba de correlación de Spearman *mediana (percentil 25–75).

CQR: Cuestionario de cumplimiento en reumatología; BMQ-T: Cuestionario turco sobre creencias acerca de los medicamentos.

En resumen, las creencias de los pacientes reumatológicos sobre los medicamentos afectaban negativamente a la adherencia, y el género, el estado civil y la situación laboral influían en la adherencia y las creencias; un uso más prolongado de los medicamentos se asociaba con creencias más sólidas, y una mayor ansiedad relacionada con los medicamentos se vinculaba con una menor adherencia, lo que pone de relieve la compleja relación entre las características de los pacientes, sus creencias y la adherencia en la atención reumatológica.

## DISCUSIÓN

En el estudio, se determinó que la duración del diagnóstico era en su mayoría (60,8%) de 5 años o más. En el estudio realizado en España^(19)^, se encontró que la duración media de la enfermedad era de 8 años. De manera similar, en el estudio realizado en Turquía^(4)^, la duración media observada de la enfermedad fue de 8 años. Dado que las enfermedades reumatológicas son crónicas, es un hecho conocido que los pacientes han sido diagnosticados mucho tiempo antes.

En nuestro estudio, se observó que el 40.2% de los pacientes padecían una enfermedad crónica concomitante, siendo la más frecuente la hipertensión (14,8%). En paralelo con nuestro hallazgo, el 31,9% de los pacientes informaron de enfermedades crónicas adicionales en el estudio de Omair et al.^(20)^. La hipertensión (20,2%) y la diabetes mellitus (17,2%) se encontraban entre las comorbilidades más comunes.

En el estudio se observó que existía una diferencia significativa en el cumplimiento del tratamiento según el género, y que el cumplimiento era mayor en los hombres. De forma similar a nuestro hallazgo, un estudio realizado con pacientes con artritis reumatoide (AR) en Nigeria^(12)^ informó de que el género masculino se asociaba con el cumplimiento. A diferencia de estos hallazgos, un estudio sobre pacientes con AR en Kerman-Shah, Irán^(21)^, informó de que el género no se asociaba con el cumplimiento del tratamiento. Se sabe que la menopausia y las situaciones estresantes provocan cambios hormonales en las mujeres. Además, los factores hormonales femeninos desempeñan un papel importante en la patogénesis de la AR^(22)^. Las fluctuaciones en los niveles de estrógeno y progesterona pueden influir tanto en la actividad de la enfermedad como en el estado psicológico, provocando fatiga, alteración de la percepción del dolor y cambios de humor^(23)^. Estos efectos fisiológicos y psicosociales pueden reducir la adherencia a regímenes de tratamiento complejos. Además, dado que las hormonas femeninas desempeñan un papel importante en la patogénesis y la gravedad de la AR, pueden afectar indirectamente a la adherencia al tratamiento^(24)^. Por lo tanto, la mayor adherencia observada en los hombres en nuestro estudio puede explicarse en parte por estos factores.

Se encontró una diferencia estadísticamente significativa entre el diagnóstico médico y la adherencia al tratamiento (p < 0,05). En una tesis de maestría en Turquía^(11)^, se observa que el 63% de las personas con esclerodermia tienen un nivel medio de adherencia al tratamiento según la “Escala de adherencia al tratamiento de Morisky”. Se determinó que la adherencia a la medicación de los pacientes con fiebre mediterránea familiar era buena^(25)^. La diversidad de la escala y el diagnóstico médico utilizados para medir la adherencia afecta a la evaluación del estado de adherencia del individuo.

En el estudio realizado en pacientes con AR de Arabia Saudita, el desempleo se asoció con una mejor adherencia^(20)^. Este hallazgo es similar al de nuestro estudio. En este estudio, la puntuación de ajuste reumatológico de los pacientes desempleados fue superior a la de los pacientes empleados. Se puede decir que las personas sin empleo tienen una mayor adherencia al tratamiento debido a situaciones como el tiempo dedicado al trabajo, los factores ambientales, el olvido de tomar la medicación debido a la intensidad del trabajo y el estrés laboral.

La puntuación total media del CQR-T fue de 48,21 ± 5,55. Se determinó que todos los pacientes incluidos en el estudio eran no cumplidores (puntuación CQR ≤80%), ya que, según el CQR, la falta de adherencia se define como una puntuación igual o inferior a 80^(13,15)^. Otro estudio realizado en Turquía^(15)^ encontró que la puntuación media del CQR era de 67,4 ± 11,7 en pacientes con fiebre mediterránea familiar. En este estudio, la tasa de pacientes no adherentes según el CQR se determinó en un 83,8%. En el estudio de pacientes con espondilitis anquilosante, la puntuación media del CQR fue de 73,59^(4)^. Un total del 64,6% fue evaluado como no adherente (puntuación CQR ≤80%), mientras que el 35,4% fue evaluado como concordante (puntuación CQR >80%). En un estudio sobre una población con artritis reumatoide en Kenia^(26)^, la puntuación media del CQR-5 se determinó en un 79% y se observó que la adherencia de los pacientes era del 49,5%. Al analizar la literatura, se han utilizado diferentes escalas para evaluar la adherencia en pacientes reumatológicos. Una de ellas es la Escala de Adherencia a la Medicación de Morisky^(27)^. Mientras que esta escala evalúa la adherencia al tratamiento, el CQR es un cuestionario desarrollado específicamente para evaluar la adherencia al tratamiento de la enfermedad en personas con enfermedades reumatológicas^(13)^. En este estudio, se incluyó más de un grupo de diagnóstico reumatológico en el ámbito de aplicación y se utilizó un centro de atención terciaria para recolectar los datos.

No se encontró una relación significativa entre la duración del diagnóstico y la adherencia al tratamiento en este estudio (p > 0,05). Sin embargo, se observó que la duración de la enfermedad contribuía significativamente a unas medidas de adherencia más elevadas^(12)^. Se cree que la diferencia en el diagnóstico médico influye en esta situación.

En el estudio de pacientes diagnosticados con AR^(28)^, se observó que el deterioro del estado funcional y el dolor que se producía con el aumento del número de enfermedades crónicas afectaban negativamente a la adherencia al tratamiento. En este estudio, no se encontró una correlación significativa entre el uso de métodos no farmacológicos, el daño permanente al organismo y las enfermedades crónicas adicionales y las creencias sobre los medicamentos y la adherencia al tratamiento.

No se encontraron diferencias significativas entre el estado civil, la duración de la enfermedad, la duración del uso de medicamentos, el uso de métodos no farmacológicos, el estado permanente en el organismo, las enfermedades crónicas adicionales y la adherencia al tratamiento. Se encontraron resultados similares en otros estudios^(21)^.

En este estudio, se encontró que la puntuación total media del BMQ era de 47,66 ± 9,58. En un estudio de pacientes con AR en Irak^(29)^, la puntuación total del BMQ se determinó en 59,3 ± 6,9.

Se observó que, a medida que aumentaba la duración del uso de medicamentos, disminuía la creencia general sobre los daños de los medicamentos y los pacientes se familiarizaban más con sus tratamientos^(29)^. En este estudio, se encontró una relación estadísticamente significativa entre la duración del uso de medicamentos y las creencias sobre los mismos. A medida que aumenta la duración del uso de medicamentos, aumenta el estado de creencia.

Determinar las actitudes y creencias sobre el tratamiento es muy importante para garantizar la adherencia al mismo. En este estudio, se encontró una correlación débil, negativa y significativa entre el CQR y el BMQ. Al analizar las subdimensiones, se determinó que existía una correlación negativa moderada entre el CRQ y la preocupación por el uso de medicamentos. A medida que aumenta el nivel de preocupación de las personas por el uso de medicamentos, disminuye su adherencia. De manera similar, en Turquía se encontró una correlación negativa entre las puntuaciones del BMQ y el CQR; a medida que aumenta la creencia de que la colchicina es necesaria, aumenta la adherencia^(15)^. Además, se demostró que la adherencia estaba significativamente asociada con la creencia de la necesidad y la preocupación por el tratamiento farmacológico^(30)^. En un estudio realizado en Marruecos^(31)^ se encontró una correlación significativa entre la puntuación del CQR y la del BMQ. El estudio sobre pacientes con Behçet encontró que, por el contrario, todas las subescalas del BMQ, excepto la puntuación general de uso excesivo, mostraban una correlación fuerte y significativa con el CQR^(32)^. En resumen, estos estudios muestran que las creencias sobre la necesidad y los daños de la medicación y la preocupación por los efectos secundarios son predictores de la adherencia. Según los resultados de nuestro estudio, a medida que aumenta la preocupación de las personas por el uso de medicamentos, se produce un efecto inverso en su adherencia al tratamiento.

Nuestros resultados ponen de relieve la importancia crucial de incorporar las creencias y preocupaciones de los pacientes relacionadas con la medicación en la atención reumatológica estándar. Las enfermeras y otros profesionales sanitarios deben emplear estrategias de comunicación centradas en el paciente que exploren y aborden los conceptos erróneos, las preocupaciones y las ansiedades sobre el uso de medicamentos a largo plazo. Los programas educativos adaptados a las características sociodemográficas de los pacientes, como el género, el estado civil y la situación laboral, pueden mejorar la adherencia al reducir las preocupaciones sobre los efectos secundarios y reforzar la percepción de la necesidad del tratamiento^(33)^. A nivel de políticas, la identificación temprana de los pacientes con riesgo de no adherencia puede reforzarse integrando la evaluación de las creencias sobre la medicación y las evaluaciones psicosociales en las vías de atención reumatológica rutinarias.

Desde el punto de vista de la salud laboral, los programas de apoyo a la salud en el lugar de trabajo y los planes flexibles de gestión de la medicación pueden ser beneficiosos, ya que las personas empleadas suelen enfrentarse a mayores obstáculos para la adherencia debido a las limitaciones de tiempo y al estrés relacionado con el trabajo. Las pruebas sugieren que los servicios integrados de salud y farmacia en el lugar de trabajo mejoran significativamente la adherencia a la medicación en comparación con los servicios comunitarios^(34)^. Estas implicaciones son especialmente relevantes para la práctica reumatológica en Turquía, donde la carga de las enfermedades crónicas es elevada y los factores relacionados con el trabajo pueden influir en los patrones de adherencia.

### Limitaciones del Estudio

Una limitación notable de este estudio es su diseño unicéntrico, realizado en un hospital de atención terciaria. Esto puede limitar la generalización de los resultados a entornos de práctica clínica más amplios o a poblaciones reumatológicas más heterogéneas, ya que la demografía de los pacientes, la disponibilidad de recursos y los protocolos institucionales en los centros terciarios pueden diferir de los de los hospitales comunitarios u otros entornos sanitarios. No se analizaron los trastornos psicológicos como la depresión o la ansiedad.

Dado que la evaluación de la presencia de depresión y ansiedad puede afectar a los conceptos de adaptación a la enfermedad y creencias, su evaluación enriquecería el contenido del estudio para obtener información más completa.

## CONCLUSIÓN

Las creencias de la persona sobre la enfermedad y los medicamentos utilizados ocupan un lugar muy importante en la evaluación de la adherencia al tratamiento relacionado con las enfermedades crónicas. Para respaldar la relación entre las creencias y el cumplimiento, se debe evaluar a la persona en la dimensión sociopsicológica. El género, el estado civil, la situación laboral y la duración del uso de la medicación son algunos de los parámetros que deben tenerse en cuenta en términos de adherencia. La preocupación por el medicamento afecta a la adherencia del individuo. Tanto el médico como la enfermera deben evaluar el grado de conciencia del individuo sobre el uso de la medicación. Se deben preguntar los efectos, los efectos secundarios, la dosis y la frecuencia de uso de los medicamentos, y esta información debe incluirse en la educación del paciente.

## Data Availability

El conjunto de datos que respaldan las conclusiones de este estudio está disponible previa solicitud al autor correspondiente.
